# Influence of oral health on frailty in patients with type 2 diabetes aged 75 years or older

**DOI:** 10.1186/s12877-022-02841-x

**Published:** 2022-02-19

**Authors:** Masaki Ishii, Yasuhiro Yamaguchi, Hironobu Hamaya, Yuko Iwata, Kazufumi Takada, Sumito Ogawa, Mitsuo Imura, Masahiro Akishita

**Affiliations:** 1 The Department of Geriatric Medicine, The University of Tokyo, 7-3-1 Hongo, Bunkyo-ku, 1138655 Tokyo, Japan; 2 Department of Pulmonary Medicine, Saitama Medical Center, Jichi Medical University, 1- 847, Amanumacho, Omiya-ku, Saitama Saitama City, Japan; 3 Okamoto Internal Medicine Clinic, 6-14-8, Shizuoka Honmachi, Yaizu City, Japan

**Keywords:** Frailty, Oral frailty index-8, Older adults, Oral health, Type 2 diabetes

## Abstract

**Background:**

Poor oral health conditions are known to affect frailty in the older adults. Diabetes is a risk factor for both poor oral health and frailty, therefore, oral health status may affect frailty in diabetic patients more than in the general population. The purpose of this study was to evaluate the influence of oral health and other factors on frailty and the relationship among oral health, diabetes and frailty in older adult patients with type 2 diabetes.

**Methods:**

Patients with type 2 diabetes aged 75 years or older were included in this cross-sectional study. Eligible patients were surveyed by questionnaire for frailty, oral health status, and cognitive and living functions. Factors influencing pre-frailty, frailty, and individual frailty screening index (FSI) classes were evaluated.

**Results:**

Of the 111 patients analyzed, 66 cases (59.5%) were categorized as robust, 33 cases (29.7%) as pre-frailty, and 12 cases (10.8%) as frailty. The oral frailty index, the cognitive and living functions score, and BMI were found to be factors influencing pre-frailty or frailty. In the evaluation of individual FSI classes, BMI had an influence on those with a FSI ≤2. The cognitive and living functions score was a factor influencing those with FSI ≤3. The oral frailty index was found to have a significant influence on all FSI classes.

**Conclusions:**

Poor oral health has an influence on frailty in patients with type 2 diabetes aged ≥75. In this patient population, as frailty progresses, the impact of oral health on frailty may increase.

**Trial registration:**

This study was retrospectively registered in UMIN-CTR (UMIN000044227).

## Background

Frailty is a condition in which physical and mental vulnerability develops with aging and has a great impact on the health and quality of life of older adults. There are various risk factors for frailty, such as underlying disease, nutritional status, and sarcopenia, which interact with each other to develop vulnerability [1]. Poor oral health conditions are known to affect frailty in the older adults. Kamden et al. reported that decreased masticatory function was a significant influential factor for frailty [2]. Tanaka et al. also reported that poor oral health status based on evaluation of masticatory function, tongue pressure, salivation, and so on, was a significant risk factor for frailty [3]. It has been suggested that the influence of poor oral health on frailty involves tooth loss, decreased salivation, periodontal disease, and dental caries [4]. Loss of teeth reduces masticatory function and worsens nutritional status [5, 6]. Saliva secretion is reduced by aging, medication such as anti-hypertensives and anti-depressants, or radiation therapy [4], and the decrease in saliva is likely to cause dental caries, leading to decreased chewing and swallowing functions [7–9].

Poor glycemic control in patients with diabetes has been reported to exacerbate various oral health conditions including dental caries, decreased salivation and periodontal disease [10]. Izuora et al. reported that in diabetic patients averaged 58.9 years old, only 6.4% had their original 32 teeth and 15.3% had lost all of their teeth [11]. The prevalence of periodontal disease is higher in diabetics than in the general population [12]. Periodontal disease also causes systemic inflammation and adversely affects diabetes through insulin resistance [13]. In addition, 46% of diabetic patients had dry mouth caused by decreased salivation [14]. These various changes in the oral cavity of diabetic patients interact with each other, resulting in deterioration of masticatory and swallowing function.

Diabetes itself is reportedly a risk factor for frailty [15–17]. Insulin is involved in promoting protein synthesis and suppressing proteolysis but, with diabetes, insulin resistance suppresses protein synthesis, affecting muscle strength and motor function [18, 19]. Diabetes has been shown to be a risk factor for both poor oral health and frailty [20], and, therefore, oral health status may affect frailty in diabetic patients more than in the general population.

The impact of nutritional status on health may vary with patient age. In Japan, the older adults aged 75 and over are termed the ‘late-stage older adults’ in the medical system. In a study of diabetic patients in Japan, Yamaoka et al. found that reduced protein intake was a significant risk factor for all-cause mortality only in patients aged 75 years and older, and thus adequate nutrition is required especially for patients aged 75 and older [21]. A strict diet to prevent obesity is important for younger patients with diabetes, but prevention of frailty and sarcopenia becomes more important as they age [22]. Considering the above, the influence of oral health on frailty may differ depending on the age and the severity of frailty. We set the hypothesis that the impact of poor oral health on frailty would increase as the frailty progresses, and conducted the study to assess the association of poor oral health with frailty in older adults with diabetes who are more likely to be affected by poor oral health.

## Materials and methods

### Design and patients

This study was conducted to evaluate the influence of oral health and other factors on frailty in older adult patients with type 2 diabetes. This is a cross-sectional study of data from Okamoto Internal Medicine Clinic, Shizuoka, Japan, collected from December 2019 to March 2020. The subjects of this study were patients with type 2 diabetes aged 75 years and older and those with gait disturbance were excluded.

### Methods

**Fig. 1:**
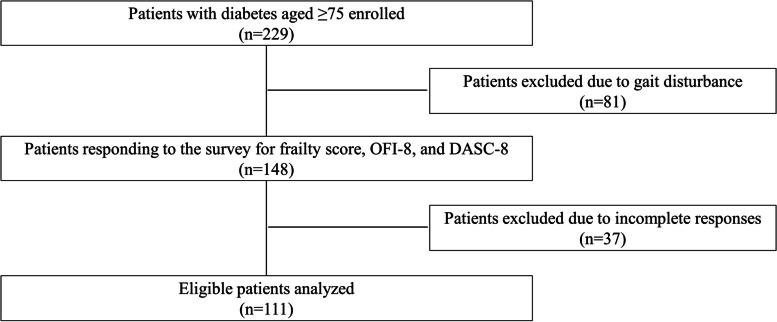
Patient inclusion/exclusion flowchart. DASC-8: the dementia assessment sheet for community-based integrated care system-8 items, OFI-8: oral frailty index-8

Of the 229 patients enrolled, 111 eligible patients were surveyed by questionnaire for frailty, oral health status, and cognitive and living functions (Fig. 1). To evaluate frailty, oral health status, and cognitive and living functions, we used the Frailty Screening Index (FSI) [23], the Oral Frailty Index-8 (OFI-8) [24], and the Dementia Assessment Sheet for Community-based Integrated Care System-8 (DASC-8) [25], respectively. Data for age, sex, BMI, duration of diabetes (date of first diagnosis of type 2 diabetes to time of investigation), and HbA1c were collected. Factors influencing frailty was examined and the adjusted odds ratios with a 95%CI of various influential factors to frailty were estimated. Adjusted odds ratios were calculated for pre-frailty, frailty, and individual FSI classes. In addition, factors influencing oral health were examined.

The OFI-8 score was the sum of the scores for each of the following questions. Q1: Do you have any difficulties eating tough foods compared to 6 months ago? (2 points for Yes), Q2: Have you choked on your tea or soup recently? (2 points for Yes), Q3: Do you use dentures? (2 points for Yes), Q4: Do you often have a dry mouth? (1 point for Yes), Q5: Do you go out less frequently than you did last year? (1 point for Yes), Q6: Can you eat hard foods like squid jerky or pickled radish? (1 point for No), Q7: How many times do you brush your teeth in a day? (3 or more times/day) (1 point for No), and Q8: Do you visit a dental clinic at least annually? (1 point for No) [24]. OFI-8 score is a discrete variable in the range of 0-11.

The FSI, a self-evaluation questionnaire developed by Yamada et al. consists of questions about five items—weight loss, gait speed, exercise frequency, short-term memory, and feeling of fatigue [23]. It was developed for the purpose of determining frailty status without measuring gait speed or grip strength [23]. The FSI was the sum of the scores for each of the following questions. Q1: Have you lost 2 kg or more in the past 6 months? (1 point for Yes), Q2: Do you think you walk slower than before? (1 point for Yes), Q3: Do you go for a walk for your health at least once a week? (1 point for No), Q4: Can you recall what happened 5 min ago? (1 point for No), Q5: In the past 2 weeks, have you felt tired without a reason? (1 point for Yes) [23]. Cases corresponding to score 0, 1 to 2, and 3 or more were defined as robust, pre-frailty, and frailty.

Cognitive and living functions were evaluated using DASC-8 developed by Toyoshima et al. [25]. Patients were classified into three categories (I, II, and III) determined by DASC-8 score—I: normal cognitive function and activities of daily living (ADL) independence; II: mild cognitive impairment or mild dementia or instrumental ADL decline, and basic ADL independence; and III: moderate or severe dementia or decreased basic ADL or many comorbidities or dysfunctions [25]. All questionnaire surveys were conducted by person-to-person interviews. In cases of patients with cognitive decline, family members were allowed to attend the interview and provide answer to the questions when necessary.

### Statistics

Descriptive statistics are expressed as n (%) and mean ± SD. The Kruskal-Wallis test was used to compare continuous and discrete variables, and the chi-square test was used to compare categorical variables. For the evaluation of the influencial factors for the FSI and frailty categories, multiple regression and multiple logistic analyses were performed on the following variables extracted using a stepwise method: sex, age, duration of diabetes, HbA1c, OFI-8 score, DASC-8 score, and BMI. In addition to oral health [2, 3], diabetes [19], cognitive function [26], BMI [27–29], and other variables reported to associate with frailty, sex and age showed a significant difference between the frailty groupings in our study (Table 1) and, therefore, were selected as independent variables. Furthermore, the scores for factors influencing frailty were converted into deviation values, and the mean deviation value was calculated for each FSI class. The numerical change in the mean deviation value for each factor influencing frailty in the FSI ranges of 0 to 4 were calculated. For the evaluation of the influential factors for the OFI-8 scores, multiple regression analysis was conducted on the following variables extracted using the stepwise method: sex, age, BMI, duration of diabetes, HbA1c, and DASC-8. All of these variables have been reported to have an effect on oral health [10, 26–29]. For the influence of diabetic duration on oral health, an OFI-8 score ≥4 was defined as oral frailty [24] and a cutoff value was calculated using receiver operating characteristic (ROC) analysis. The two-sided α was set to be 0.05. Statistical analysis was performed using SPSS (IBM).

**Table 1:** Patient characteristics

		Total (*n *=111)	Robust (*n *= 66)	Pre-frailty (*n *= 33)	Frailty (*n *= 12)	p
Sex (male), n (%)		72 (64.9%)	49 (74.2%)	18 (54.5%)	5 (41.7%)	0.032
Age (y.o.), mean±SD		79.7 ± 3.8	78.8 ± 3.1	80.7 ± 4.4	82.3 ± 3.5	0.004
BMI, mean±SD		23.2 ± 3.8	22.8 ± 3.8	23.9 ± 4.2	23.3 ± 2.5	0.336
Duration of diabetes (years), mean±SD		20.4 ± 11.1	19.7 ± 10.5	19.5 ± 10.6	26.4 ± 14.5	0.182
≤20 years, n (%)		57 (51.4%)	35 (53.0%)	19 (57.6%)	3 (25.0%)	0.131
>20 years, n (%)		54 (48.6%)	31 (47.0%)	14 (42.4%)	9 (75.0%)	
HbA1c (%), mean±SD		7.1 ± 0.7	7.1 ± 0.6	7.3 ± 0.9	7.2 ± 0.6	0.615
Frailty screening index, mean±SD		0.8 ± 1.1	0 ± 0	1.5 ± 0.5	3.3 ± 0.5	<0.001
OFI-8 score, mean±SD		3.9 ± 2.0	3.3 ± 1.8	4.4 ± 1.8	5.8 ± 2.4	<0.001
Score <4, n (%)		52 (46.8%)	39 (59.1%)	11 (33.3%)	2 (16.7%)	0.003
Score ≥4, n (%)		59 (53.2%)	27 (40.9%)	22 (66.7%)	10 (83.3%)	
DASC-8, mean±SD		10.2 ± 3.0	9.2 ± 1.3	11.1 ± 3.7	13.6 ± 4.4	<0.001
Category I, n (%)		80 (72.1%)	57 (86.4%)	19 (57.6%)	4 (33.3%)	-
Category II, n (%)		25 (22.5%)	9 (13.6%)	11 (33.3%)	5 (41.7%)	
Category III, n (%)		6 (5.4%)	0	3 (9.1%)	3 (25.0%)	

*DASC-8* the dementia assessment sheet for community-based integrated care system-8 items, *OFI-8* score oral frailty index-8

## Results

### Patient characteristics

**Fig. 2:**
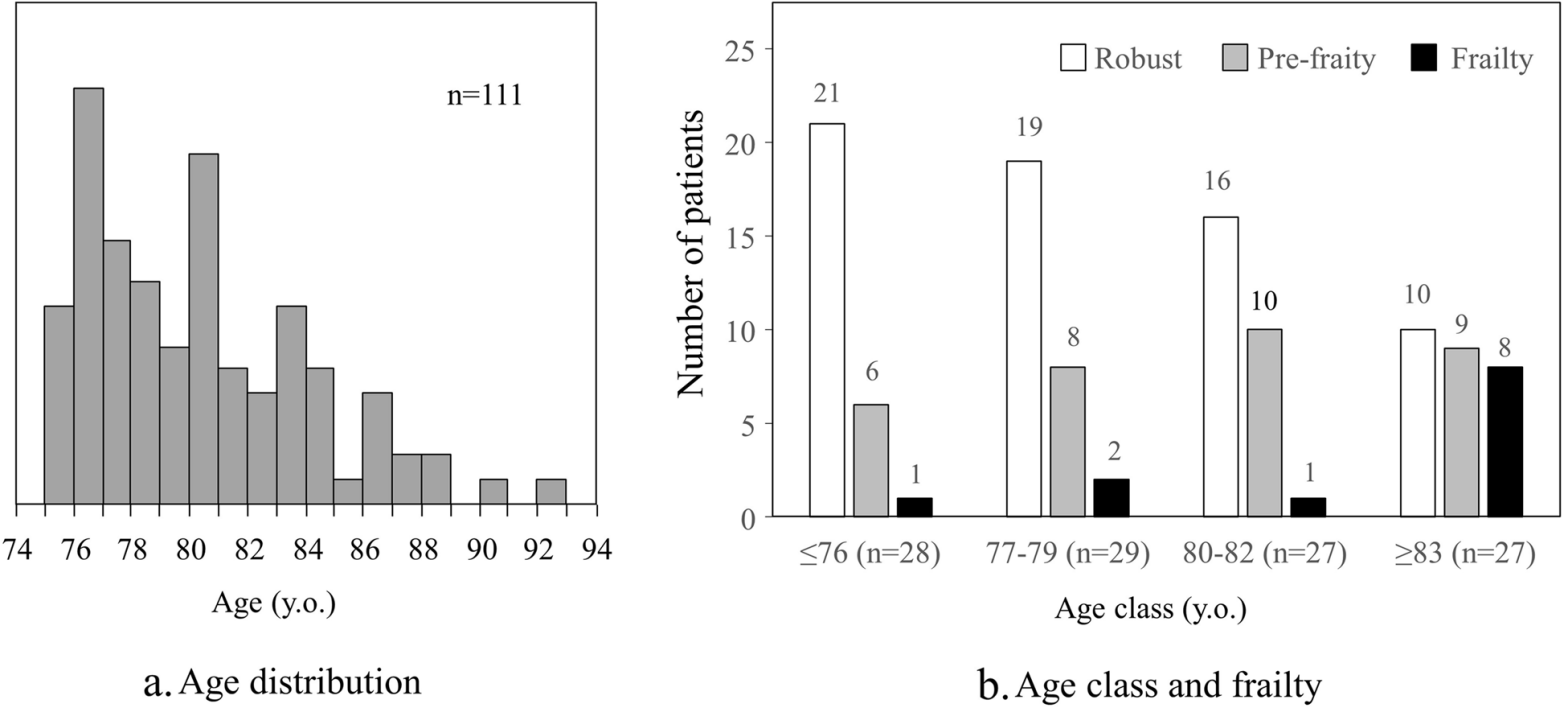
Age distribution and rates of frailty

Of the patients treated for type 2 diabetes from December 2019 to March 2020, 229 patients aged 75 years and older were enrolled; 81 were excluded because of gait disturbance (Fig. 1). Questionnaire surveys on OFI-8, FSI, and DASC-8 were conducted in 148 of the patients. Of those, 37 patients were excluded due to incomplete answers, and the remaining 111 patients were analyzed. The age range was 75 to 92 years, with the number of patients decreasing as the age increased (Fig. 2a). The absolute number of patients with frailty or pre-frailty increased in the older population, with 67% of frailty patients aged 83 and over (Fig. 2b). The mean age was 79.7 years, with an average duration of diabetes of 20.4 years, of which 54 (48.6%) had a duration of diabetes of 20 years or more (Table 1). Regarding frailty status, 66 (59.5%) cases were defined as robust, 33 (29.7%) cases as pre-frailty, and 12 (10.8%) as frailty. The rate for each category of the DASC-8 was 72.1% (I), 22.5% (II) and 5.4% (III). Significant differences were found in sex, age, OFI-8 score, and DASC-8 score in multiple comparisons among the robust, pre-frailty, and frailty groups.

### Factors influencing frailty

**Fig. 3:**
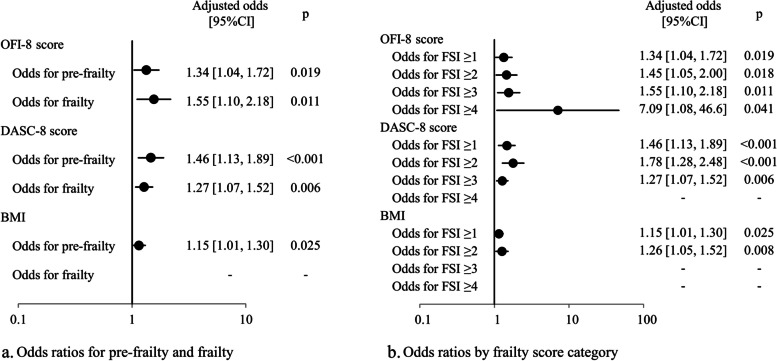
Adjusted odds ratios for frailty. DASC-8: the dementia assessment sheet for community-based integrated care system-8 items, FSI: frailty screening index, OFI-8: oral frailty index-8

Multiple regression analysis showed that OFI-8 score, DASC-8, and BMI were factors associated with increased FSI (Table 2). Multiple logistic analysis showed that OFI-8 score, DASC-8 score, and BMI were factors influencing pre-frailty, and that OFI-8 score and DASC-8 were factors influencing frailty. The adjusted odds with 95% confidence interval [95%CI] of the OFI-8 scores were 1.34 [1.04, 1.72] for pre-frailty and 1.55 [1.10, 2.18] for frailty (Fig. 3a). In the evaluation by individual FSI classes, BMI was not found to be a factor influencing a FSI ≥3 (Fig. 3b). The DASC-8 score was not a factor influencing a FSI ≥4. The OFI-8 score was a significant factor influencing all FSI classes. The adjusted odds ratios with 95%CI of OFI-8 scores were 1.34 [1.04, 1.72] for FSI ≥1, 1.45 [1.05, 2.00] for FSI ≥2, 1.55 [1.10, 2.18] for FSI ≥3 and 7.09 [1.08, 46.6] for FSI ≥4.

**Table 2:** Factors influencing frailty screening index, pre-frailty, frailty, and oral frailty score

Factors influencing frailty screening index, model: *p* < 0.0001					
Variable		Estimate	S.E.	t value	p
Sex (male)		-0.12	0.09	-1.31	0.1936
Age		0.04	0.02	1.62	0.1078
HbA1c		0.15	0.12	1.25	0.2143
OFI-8 score		0.14	0.04	3.13	0.0022
DASC8		0.15	0.03	4.94	<0.0001
BMI		0.05	0.02	2.28	0.0245
Factors influencing pre-frailty, model: *p *< 0.001					
Variable		Estimate	S.E.	Chi-square	p
Sex (male)		-0.40	0.26	2.43	0.119
Age		0.08	0.07	1.33	0.2489
OFI-8 score		0.29	0.13	5.04	0.0248
DASC-8		0.38	0.13	8.57	0.0034
BMI		0.14	0.06	4.67	0.0307
Factors influencing frailty, model: *p* < 0.0001					
Variable		Estimate	S.E.	Chi-square	p
OFI-8 score		0.44	0.17	6.41	0.0114
DASC-8		0.24	0.09	7.52	0.0061
Factors influencing OFI-8 score, model: *p* = 0.0024					
Variable		Estimate	S.E.	t value	p
Sex (male)		-0.35	0.19	-1.85	0.0673
Age		0.09	0.052	1.75	0.0835
Duration of diabetes		0.039	0.016	2.45	0.0159
HbA1c		-0.30	0.25	-1.17	0.2446
DASC-8		0.087	0.066	1.33	0.1868

*DASC-8* the dementia assessment sheet for community-based integrated care system-8 items, *OFI-8* oral frailty index-8

**Fig. 4:**
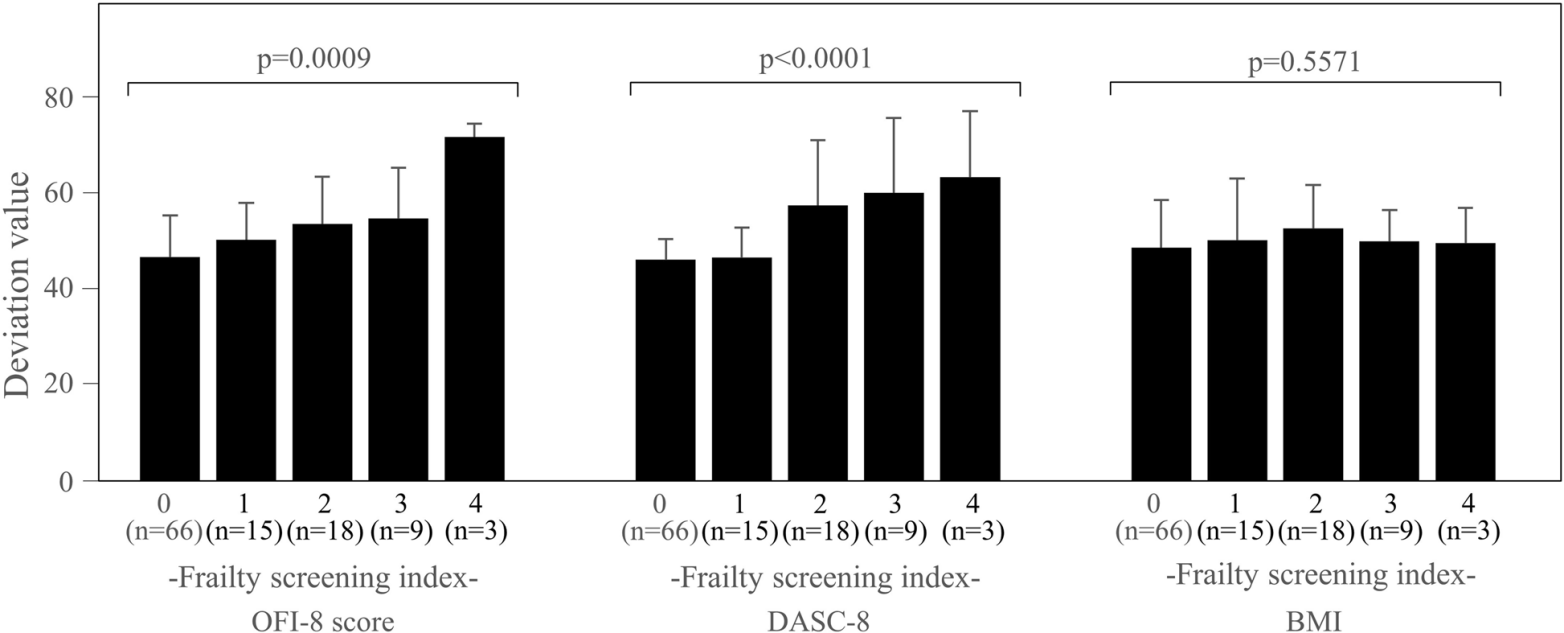
Frailty screening index and deviation values for OFI-8, DASC-8, and BMI

Figure 4 shows the deviation values of OFI-8, DASC-8, and BMI by FSI classes. The deviation values of OFI-8 and DASC-8 increased as the FSI increased (*p* = 0.0009 and *p* < 0.0001), but the deviation values of BMI did not clearly show change (*p* = 0.5571). The maximum change in the mean deviation value for the range of FSI 0 to 4 was greatest with 25.2 for OFI-8, followed by 17.3 for DASC-8 and 1.0 for BMI.

### Factors influencing OFI-8 score

**Fig. 5:**
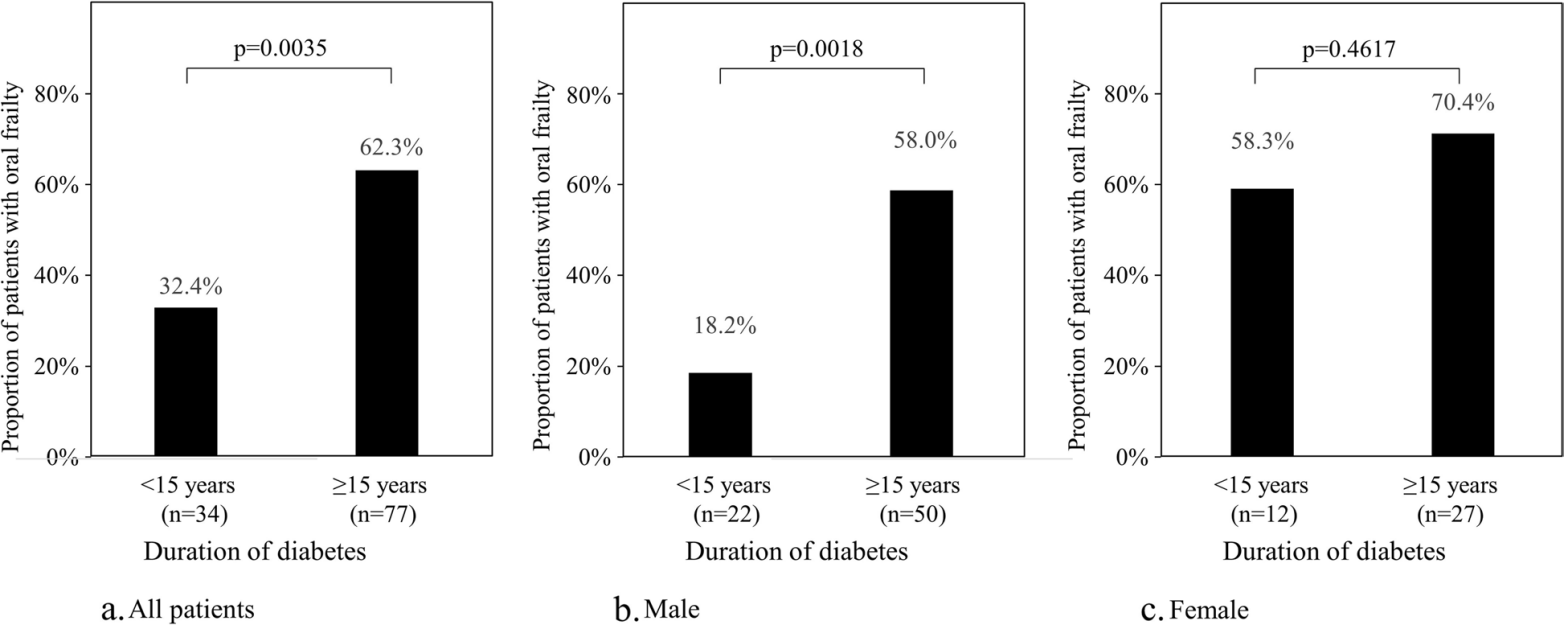
Duration of diabetes and proportion of patients with oral frailty. Oral frailty was defined as an oral frailty score ≥4

Multiple regression analysis showed that duration of diabetes was a significant influencing factor on OFI-8 score (Table 2). The cut-off value of the duration of diabetes for oral frailty from ROC analysis was 15 years (AUC: 0.63). The proportion of patients with oral frailty was 32.4% in duration of diabetes <15 years and 62.3% in that of ≥15 years (Fig. 5a). The proportion of patients with oral frailty by sex and duration of diabetes were 18.2% (<15 years) vs. 58.0% (≥15 years) in male and 58.3% vs. 70.4% in female (Fig. 5b and c).

## Discussion

The influence of oral health on frailty was investigated in 111 patients with type 2 diabetes aged 75 years and older. Frailty was found in 10.8% (12/111), 67% of whom were aged 83 years or older. OFI-8 score, DASC 8 score, and BMI were all factors influencing FSI, but the impact of these variables differed depending on the severity of frailty. A higher OFI-8 score was a significant factor influencing all FSI classes.

In the older adults, poor oral health represented by deterioration of masticatory function and swallowing function has been shown to affect sarcopenia and frailty [4, 30]. Older adults with impaired masticatory function often avoid eating hard-to-eat foods such as meat, fruits, and vegetables [31–33], and these changes in eating habits increase the risk of malnutrition and frailty [30, 34, 35]. Our results support the results of these previous studies. To our knowledge, this is the first report showing the relationship between oral health and frailty in diabetic patients aged ≥75.

In this study, OFI-8 score, DASC-8 score and BMI were shown to be factors influencing pre-frailty or frailty. High BMI has been reported to be a risk factor for frailty [27–29]. Excessive lipid deposition and infiltration of lipids into muscle fibers affect muscle weakness and activity [36], and excessive lipid deposition has been suggested to increase the risk of frailty through systemic inflammation [37]. However, in our study, BMI showed no significant effect on frailty or FSI ≥3. As mentioned above, previous studies have shown that BMI was a risk factor for frailty [27–29], but the studies by Niederstrasser et al. and Hubbard et al. were conducted in subjects aged ≥50 [27, 29], and the study by Watanabe et al. was conducted in subjects aged ≥65 [28]. Our study was conducted in patients with diabetes aged ≥75, a population at higher risk of frailty than those in previous studies. Considering the above, the effect of BMI on frailty may vary depending on patient age and severity of frailty.

In the evaluation of individual FSI classes, BMI was not a factor influencing FSI ≥3 and DASC-8 was not a factor influencing FSI ≥4, but the OFI-8 score was consistently a significant factor influencing all classes of FSI. Furthermore, in the evaluation of the data converted into deviation values, we found that when the FSI change from 0 to 4, the change in the deviation value was the largest for OFI-8 data, and no significant change was observed in the deviation value for BMI. These results indicate that as frailty progresses, the impact of oral health on frailty increases and the effects of BMI and DASC-8 show a relative decrease.

In our study, the cut-off value for the duration of diabetes for oral frailty, defined as OFI-8 score ≥4, [24] was 15 years. The proportions of patients with oral frailty by sex and duration of diabetes were 18.2% (<15 years) vs. 58.0% (≥15 years) for males and 58.3% vs. 70.4% for females, indicating that female patients develop oral frailty earlier than male patients.

The strength of this study is that this is the first study showing a relationship among diabetes, oral health, and frailty in diabetic patients aged ≥75. The weakness of this study is that only 12 patients (10.8%) were designated as frailty.

There are several limitations to this study. This was a cross-sectional study conducted in a single institution. The OFI-8 score as an index of oral health is based on a questionnaire, and oral function including masticatory function and swallowing function was not evaluated by dental or oral specialists. Energy and protein intake that affects frailty and status of diabetes treatment was not considered in this study. The number of patients was 111 and so there is a limitation to the extent of the analysis. The subjects of this study were older adults aged 75 and older and so the influence of memory bias cannot be ruled out. Factors reported to influence frailty include age, educational history, various complications, nutritional status [1], cognitive function [26], fall history [38], and type 2 diabetes [18, 19] and so on. Of these, the independent variables used in this study were sex, age, duration of diabetes, HbA1c, oral frailty, BMI, and cognitive/living function. In this study, a stepwise method was used to select independent variables for the multivariate analysis. The subjects of the study were type 2 diabetic patients aged 75 years or older who do not have gait disturbance and so our results are not applicable to the general population. The results of this study need to be interpreted with these factors in mind.

## Conclusions

This is the first study to show that poor oral health increases the risk of frailty in diabetic patients aged 75 years and older. Oral health is a factor influencing frailty, and as frailty progresses, the impact of oral health on frailty may increase.

## Data Availability

The datasets used during the current study are available from the corresponding author on reasonable request.

## Abbreviations

ADL: activities of daily living
DASC-8: Dementia Assessment Sheet for Community-based Integrated Care System-8
OFI-8: oral frailty index-8
ROC: receiver operating characteristic
